# Edible Mushrooms: Improving Human Health and Promoting Quality Life

**DOI:** 10.1155/2015/376387

**Published:** 2015-01-20

**Authors:** María Elena Valverde, Talía Hernández-Pérez, Octavio Paredes-López

**Affiliations:** Centro de Investigación y de Estudios Avanzados (IPN), Unidad Irapuato, Km 9.6 Libramiento Norte Carretera Irapuato-León, 36821 Irapuato, GTO, Mexico

## Abstract

Mushrooms have been consumed since earliest history; ancient Greeks believed that mushrooms provided strength for warriors in battle, and the Romans perceived them as the “Food of the Gods.” For centuries, the Chinese culture has treasured mushrooms as a health food, an “elixir of life.” They have been part of the human culture for thousands of years and have considerable interest in the most important civilizations in history because of their sensory characteristics; they have been recognized for their attractive culinary attributes. Nowadays, mushrooms are popular valuable foods because they are low in calories, carbohydrates, fat, and sodium: also, they are cholesterol-free. Besides, mushrooms provide important nutrients, including selenium, potassium, riboflavin, niacin, vitamin D, proteins, and fiber. All together with a long history as food source, mushrooms are important for their healing capacities and properties in traditional medicine. It has reported beneficial effects for health and treatment of some diseases. Many nutraceutical properties are described in mushrooms, such as prevention or treatment of Parkinson, Alzheimer, hypertension, and high risk of stroke. They are also utilized to reduce the likelihood of cancer invasion and metastasis due to antitumoral attributes. Mushrooms act as antibacterial, immune system enhancer and cholesterol lowering agents; additionally, they are important sources of bioactive compounds. As a result of these properties, some mushroom extracts are used to promote human health and are found as dietary supplements.

## 1. Introduction

Mushrooms have been considered as ingredient of gourmet cuisine across the globe; especially for their unique flavor and have been valued by humankind as a culinary wonder. More than 2,000 species of mushrooms exist in nature, but around 25 are widely accepted as food and few are commercially cultivated. Mushrooms are considered as a delicacy with high nutritional and functional value, and they are also accepted as nutraceutical foods; they are of considerable interest because of their organoleptic merit, medicinal properties, and economic significance [1, 2]. However, there is not an easy distinction between edible and medical mushrooms because many of the common edible species have therapeutic properties and several used for medical purposes are also edible [3].

The most cultivated mushroom worldwide is* Agaricus bisporus*, followed by* Lentinus edodes*,* Pleurotus* spp., and* Flammulina velutipes*. Mushrooms production continuously increases, China being the biggest producer around the world [1, 4, 5]. However, wild mushrooms are becoming more important for their nutritional, sensory, and especially pharmacological characteristics [2].

Mushrooms could be an alternative source of new antimicrobial compounds, mainly secondary metabolites, such as terpenes, steroids, anthraquinones, benzoic acid derivatives, and quinolones, but also of some primary metabolites like oxalic acid, peptides, and proteins.* Lentinus edodes* is the most studied species and seems to have an antimicrobial action against both gram-positive and gram-negative bacteria [6].

They have a great nutritional value since they are quite rich in protein, with an important content of essential amino acids and fiber, poor fat but with excellent important fatty acids content (Table 1). Moreover, edible mushrooms provide a nutritionally significant content of vitamins (B1, B2, B12, C, D, and E) [7, 8]. Thus, they could be an excellent source of many different nutraceuticals and might be used directly in human diet and to promote health for the synergistic effects of all the bioactive compounds present [9–13].

A large variety of mushrooms have been utilized traditionally in many different cultures for the maintenance of health, as well as in the prevention and treatment of diseases through their immunomodulatory and antineoplastic properties. In the last decade, the interest for pharmaceutical potential of mushrooms has been increased rapidly, and it has been suggested that many mushrooms are like mini-pharmaceutical factories producing compounds with miraculous biological properties [5, 14]. In addition, the expanded knowledge of the molecular basis of tumorigenesis and metastasis has given the opportunity for discovering new drugs against abnormal molecular and biochemical signals leading to cancer [15].

More than 100 medicinal functions are produced by mushrooms and fungi and the key medicinal uses are antioxidant, anticancer, antidiabetic, antiallergic, immunomodulating, cardiovascular protector, anticholesterolemic, antiviral, antibacterial, antiparasitic, antifungal, detoxification, and hepatoprotective effects; they also protect against tumor development and inflammatory processes [16–19]. Numerous molecules synthesized by macrofungi are known to be bioactive, and these bioactive compounds found in fruit bodies, cultured mycelium, and cultured broth are polysaccharides, proteins, fats, minerals, glycosides, alkaloids, volatile oils, terpenoids, tocopherols, phenolics, flavonoids, carotenoids, folates, lectins, enzymes, ascorbic, and organic acids, in general. Polysaccharides are the most important for modern medicine and *β*-glucan is the best known and the most versatile metabolite with a wide spectrum of biological activity [5, 16, 17, 20].

A balanced diet is the supporting treatment for the prevention of illness and especially against oxidative stress. In this context, mushrooms have a long history of use in the oriental medicine to prevent and fight numerous diseases. Nowadays, mushroom extracts are commercialized as dietary supplements for their properties, mainly for the enhancement of immune function and antitumor activity [3, 9, 11, 17, 21–26]. In this work, we aimed to review the nutritional value as well as the chemical and nutraceutical composition, and commercial potentialities of the most cultivated edible mushrooms worldwide.

## 2. Findings and Discussion

### 2.1. Nutritional Value

The nutritional value of edible mushrooms is due to their high protein, fiber, vitamin and mineral contents, and low-fat levels [8, 10]. They are very useful for vegetarian diets because they provide all the essential amino acids for adult requirements; also, mushrooms have higher protein content than most vegetables. Besides, edible mushrooms contain many different bioactive compounds with various human health benefits [27, 28].

It is important to remark that the growth characteristics, stage and postharvest condition may influence the chemical composition and the nutritional value of edible mushrooms. Also, great variations occur both among and within species [29, 30]. Mushrooms contain a high moisture percentage that ranges between 80 and 95 g/100 g, approximately. As above mentioned, edible mushrooms are a good source of protein, 200–250 g/kg of dry matter; leucine, valine, glutamine, glutamic and aspartic acids are the most abundant. Mushrooms are low-calorie foods since they provide low amounts of fat, 20–30 g/kg of dry matter, being linoleic (C18:2), oleic (C18:1) and palmitic (C16:0) the main fatty acids. Edible mushrooms contain high amounts of ash, 80–120 g/kg of dry matter (mainly potassium, phosphorus, magnesium, calcium, copper, iron, and zinc). Carbohydrates are found in high proportions in edible mushrooms, including chitin, glycogen, trehalose, and mannitol; besides, they contain fiber, *β*-glucans, hemicelluloses, and pectic substances. Additionally, glucose, mannitol, and trehalose are abundant sugars in cultivated edible mushrooms, but fructose and sucrose are found in low amounts. Mushrooms are also a good source of vitamins with high levels of riboflavin (vitamin B2), niacin, folates, and traces of vitamin C, B1, B12, D and E. Mushrooms are the only nonanimal food source that contains vitamin D and hence they are the only natural vitamin D ingredients for vegetarians. Wild mushrooms are generally excellent sources of vitamin D2 unlike cultivated ones; usually cultivated mushrooms are grown in darkness and UV-B light is needed to produce vitamin D2 [3, 8, 29–34].

### 2.2. Nutraceuticals

In addition to the nutritional components found in edible mushrooms, some have been found to comprise important amounts of bioactive compounds. The content and type of biologically active substances may vary considerably in edible mushrooms; their concentrations of these substances are affected by differences in strain, substrate, cultivation, developmental stage, age, storage conditions, processing, and cooking practices [8–10].

The bioactive substances found in mushrooms can be divided into secondary metabolites (acids, terpenoids, polyphenols, sesquiterpenes, alkaloids, lactones, sterols, metal chelating agents, nucleotide analogs, and vitamins), glycoproteins and polysaccharides, mainly *β*-glucans. New proteins with biological activities have also been found, which can be used in biotechnological processes and for the development of new drugs, including lignocellulose-degrading enzymes, lectins, proteases and protease inhibitors, ribosome-inactivating proteins, and hydrophobins [35].

In China, many species of edible wild-grown mushrooms, that is* Tricholoma matsutake, Lactarius hatsudake*,* Boletus aereus*, are appreciated as food and also in traditional Chinese medicine. The rich amount of proteins, carbohydrates, essential minerals, and low energy levels contributes to considering many wild-grown mushrooms as good food for the consumer, which can virtually be compared with meat, eggs, and milk [36].

Numerous bioactive polysaccharides or polysaccharide-protein complexes from medicinal mushrooms appear to enhance innate and cell-mediated immune responses and exhibit antitumor activities in animals and humans. A wide range of these mushroom polymers have been reported previously to have immunotherapeutic properties by facilitating growth inhibition and destruction of tumor cells. Several of the mushroom polysaccharide compounds have proceeded through clinical trials and are used extensively and successfully in Asia to treat various cancers and other diseases. A total of 126 medicinal functions are thought to be produced by selected mushrooms [37].

#### 2.2.1. Carbohydrates

Polysaccharides are the best known and most potent mushroom derived substances with antitumor and immunomodulating properties. Data on mushroom polysaccharides have been collected from hundreds of different species of higher basidiomycetes; some specific carbohydrates with these properties have been quantified in different mushrooms: rhamnose, xylose, fucose, arabinose, fructose, glucose, mannose, mannitol, sucrose, maltose, and trehalose (Table 2) [11, 15, 38, 39].

The antitumor polysaccharides isolated from mushrooms are acidic or neutral, with strong antitumor action and differ significantly in their chemical structures. A wide range of glycans extending from homopolymers to highly complex heteropolymers exhibits antitumoral activity. Mushroom polysaccharides have antitumor action by activation of the immune response of the host organism, in other words, mushroom polysaccharides do not directly kill tumor cells. These compounds prevent stress on the body and they may produce around 50% reduction in tumor size and prolong the survival time of tumor bearing mice [39, 40].

*β*-glucans are the main polysaccharides found in mushrooms and around half of the fungal cell wall mass is constituted by *β*-glucans. This is important for the industry because many of them are excreted into the cell growth medium, making their recovery, purification and chemical characterization very simple [41–43]. *β*-glucans are responsible for anticancer, immunomodulating, anticholesterolemic, antioxidant, and neuroprotective activities of many edible mushrooms. Also, they are recognized as potent immunological stimulators in humans, and it has been demonstrated their capacity for treating several diseases. *β*-glucans bind to a membrane receptor and induce these biological responses [44–47].

Natural products with fungal *β*-glucans have been consumed for thousands of years and they have long been considered to improve general health [48]. *β*-glucans are not synthesized by humans and they are not recognized by human immune systems as self-molecules; as a result they induce both innate and adaptive immune responses [49]. Fungal *β*-glucans are notably beneficial to humans; they markedly stimulate the human immune system and protect from pathogenic microbes and from harmful effects of environmental toxins and carcinogens that impaired immune systems. They also protect from infectious diseases and cancer and aid patients recovery from chemotherapy and radiotherapy. Besides, these compounds are also beneficial to middle-age people, people with active and stressful lifestyles, and athletes. A large variability can be observed in mushroom species and their concentration ranges from 0.21 to 0.53 g/100 g dry basis [20, 50].

*β*-glucans are well known for their biological activity, specifically related to the immune system. Hence, activating and reinforcing the host immune system seem to be the best strategy for inhibiting the growth of cancer cells [17, 51].

#### 2.2.2. Proteins

Bioactive proteins are an important part of functional components in mushrooms and also have great value for their pharmaceutical potential. Mushrooms produce a large number of proteins and peptides with interesting biological activities such as lectins, fungal immunomodulatory proteins, ribosome inactivating proteins, antimicrobial proteins, ribonucleases, and laccases [52].

Lectins are nonimmune proteins or glycoproteins binding specifically to cell surface carbohydrates and in the past few years many mushroom lectins have been discovered [53]. They have many pharmaceutical activities and possess immunomodulatory properties, antitumoral, antiviral, antibacterial, and antifungal activity. Some of them exhibit highly potent antiproliferative activity toward some tumor cell lines (human leukemic T cells, hepatoma Hep G2 cells, and breast cancer MCF7 cells) [52, 54].

Fungal immunomodulatory proteins are a new family of bioactive proteins isolated from mushrooms, which have shown a potential application as adjuvants for tumor immunotherapy mainly due to their activity in suppressing tumor invasion and metastasis [55]. Xu et al. [52] published an extensive and comprehensive review about bioactive proteins in mushrooms.

#### 2.2.3. Lipids

Polyunsaturated fatty acids are mostly contained in edible mushrooms; thus, they may contribute to the reduction of serum cholesterol. It is noteworthy that transisomers of unsaturated fatty acids have not been detected in mushrooms (Table 3) [3, 9]. The major sterol produced by edible mushrooms is ergosterol, which shows antioxidant properties [3]. It has been observed that a diet rich in sterols is important in the prevention of cardiovascular diseases [29].

Tocopherols, found in the lipidic fraction, are natural antioxidants because they act as free radical scavenging peroxyl components produced from different reactions. These antioxidants have high biological activity for protection against degenerative malfunctions, cancer, and cardiovascular diseases. Linoleic acid, an essential fatty acid to humans, takes part in a wide range of physiological functions; it reduces cardiovascular diseases, triglyceride levels, blood pressure, and arthritis [11, 30, 38, 56].

#### 2.2.4. Phenolic Compounds

Phenolic compounds are secondary metabolites possessing an aromatic ring with one or more hydroxyl groups, and their structures can be a simple phenolic molecule or a complex polymer. They exhibit a wide range of physiological properties, such as antiallergenic, antiatherogenic, anti-inflammatory, antimicrobial, antithrombotic, cardioprotective, and vasodilator effects. The main characteristic of this group of compounds has been related to its antioxidant activity because they act as reducing agents, free radical scavengers, singlet oxygen quenchers, or metal ion chelators [11, 38, 57].

Phenolic compounds provide protection against several degenerative disorders, including brain dysfunction, cancer, and cardiovascular diseases. This property is related to their capacity to act as antioxidants; they can scavenge free radicals and reactive oxygen species. The process of oxidation is essential for living organisms; it is necessary for the production of energy. However, the generation of free radicals has been implicated in several human diseases. The phenolic compounds in mushrooms show excellent antioxidant capacity [17, 58–61].

Palacios et al. [62] evaluated total phenolic and flavonoid contents in eight types of edible mushrooms (*Agaricus bisporus*,* Boletus edulis*,* Calocybe gambosa*,* Cantharellus cibarius*,* Craterellus cornucopioides*,* Hygrophorus marzuolus*,* Lactarius deliciosus*, and* Pleurotus ostreatus*). These authors concluded that mushrooms contain 1–6 mg of phenolics/g of dried mushroom and the flavonoid concentrations ranged between 0.9 and 3.0 mg/g of dried matter; the main flavonoids found were myricetin and catechin.* B. edulis* and* A. bisporus* presented the highest content of phenolic compounds, while* L. deliciosus* showed a high amount of flavonoids and* A. bisporus*,* P. ostreatus*, and* C. gambosa* presented low levels. Heleno et al. [38] reported protocatechuic,* p-*hydroxybenzoic,* p*-coumaric and cinnamic acids in the phenolic fraction in five wild mushrooms from northeastern Portugal.

### 2.3. Main Edible Mushrooms Worldwide

#### 2.3.1. Agaricus

**Figure 1:**
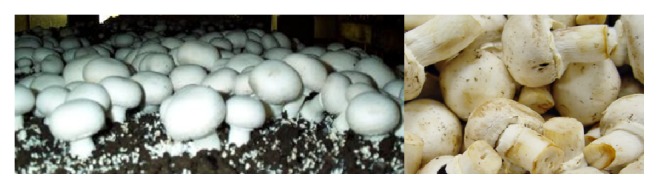
*Agaricus* species, the most cultivated mushroom worldwide.

*A. bisporus,* from the* Agaricus* genera, is the most cultivated mushroom worldwide (Figure 1). This group of edible mushrooms is nowadays widely used and studied for its medicinal and therapeutic properties [40, 63, 64].

A lectin from* A. bisporus* and a protein from* A. polytricha* have been found to be potent immune stimulants; thus, these macromolecules may be considered for pharmaceutical utilization and these fungi may be classified as healthy food.* A. bisporus* extract has been shown to prevent cell proliferation in breast cancer [5, 65, 66].

*A. blazei* is an edible mushroom native to Brazil and it has been cultivated especially in Japan. It is a very popular basidiomycete known as “sun mushroom,” and at these days it is consumed globally as food or in tea due to its medicinal properties. Its fruit bodies exhibit antimutagenic, anticarcinogenic, and immunostimulative activities [67, 68]; its extracts have also shown immunomodulatory, anticarcinogenic, and antimutagenic properties [69]. Additionally, it has been reported that this mushroom blocks the liver lipid peroxidation.

Al-Dbass et al. [70] concluded that* A. blazei* is a natural source of antioxidant compounds and has hepatoprotective activities against liver damage. On the other hand, Hakime-Silva et al. [67] reported that the aqueous extract of this fungus is a possible source of free radical scavengers and stated that this fungus can be used as a pharmacological agent against oxidative stress and as a nutritional source. Also, it is known that this fungus is rich in *β*-glucans, steroids, tocopherols, and phenolic compounds [30, 63, 71].

Moreover, liquid extracts of this fungus inhibit cell proliferation in prostate cancer cells and oral supplementation suppressing significantly tumor growth without inducing adverse effects.* A. blazei* has been used as an adjuvant in cancer chemotherapy and various types of antileukemic bioactive components have been extracted from it [5, 67].

In 2013, Carneiro et al. [22] reported powder formulations from* A. blazei* and* L. edodes* with proteins, carbohydrates, and unsaturated fatty acids. These formulations may be used in low-calorie diets and have shown high antioxidant activity with high content of tocopherols and phenolic compounds. In view of the previous studies, this fungus has been used as a healthy food for the prevention of a range of illnesses including cancer, diabetes, arteriosclerosis, and chronic hepatitis [70, 72].

*A. subrufescens* is called the “almond mushroom” for its almond taste, and it is cultivated in the US and has been incorrectly referred as* A. blazei*. It produces various bioactive compounds that have potential to treat many diseases and has been used as a medicinal food for the prevention of cancer, diabetes, hyperlipidemia, arteriosclerosis, and chronic hepatitis. Some of its beneficial properties are the reduction of tumor growth, antimicrobial and antiviral activities, immunostimulatory and antiallergy effects. The bioactive compounds isolated from this mushroom are mainly based on polysaccharides such as riboglucans, *β*-glucans, and glucomannans. The antitumor activity has been found in lipid fractions, that is, ergosterol [63, 72, 73].

#### 2.3.2. Lentinus

**Figure 2:**
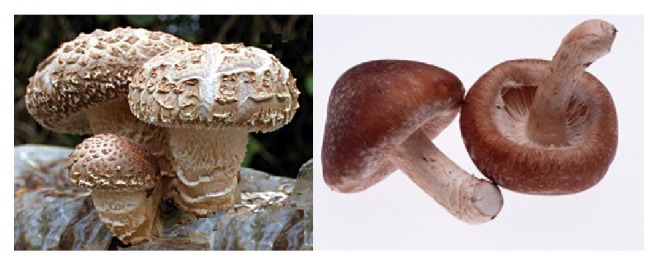
*Lentinus edodes* or “shiitake mushroom.”

*L. edodes* or “shiitake mushroom” has been used for many years to investigate functional properties and to isolate compounds for pharmaceutical use; this is because of its positive effects on human health (Figure 2). It has been utilized to alleviate the common cold for hundreds of years and some scientific evidence has supported this belief [8]. Finimundy et al. [17] have provided experimental information about the aqueous extracts of* L. edodes* as potential sources of antioxidant and anticancer compounds. These extracts significantly decreased cell proliferation on tumor as well.

Manzi and Pizzoferrato [50] reported that* L. edodes* contains high levels of *β*-glucans in the soluble fraction of dietary fiber. Shiitake produces lentinan and *β*-glucan that suppress leukemia cell proliferation and have antitumor and hypocholesterolemic activity [5, 74–78]. Lentinan is used in clinic assays as adjuvant in tumor therapy and specifically in radiotherapy and chemotherapy. On the other hand, it has been reported that lentinan enhances host resistance against infections by bacteria, fungi, parasites, and virus; it also promotes nonspecific inflammatory responses, vascular dilation, hemorrhage-inducing factors activation, and generation of helper and cytotoxic T cells [17, 74, 79, 80]. In other studies,* L. edodes* exhibited capacity to inhibit the growth of mouse sarcoma, probably due to the presence of an unspecified water-soluble polysaccharide [50].

Another edible mushroom is* L. polychrous*, found in northern and north-eastern Thailand, which is used as medicine in diseases like dyspepsia or envenomation caused by snake or scorpion. The methanolic extract and crude polysaccharides have antioxidative activity and inhibitory effect on cell proliferations of breast cancer [81–83]. Additionally, mycelial extracts from this mushroom have antiestrogenic activity, resulting from a new polyhydroxyoctane and several ergostanoids [84].

#### 2.3.3. Pleurotus

**Figure 3:**
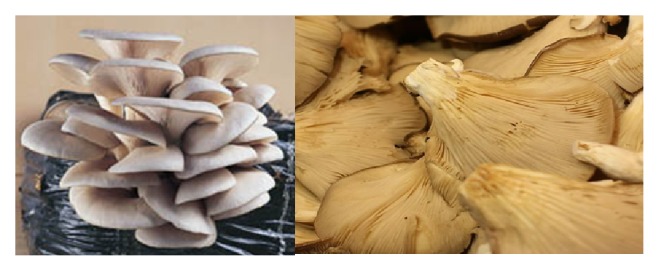
*Pleurotus* or “oyster mushroom” possesses medicinal properties and health-promoting effects.

This genus, also known as oyster mushrooms, has approximately 40 species (all are commonly edible and available) (Figure 3). In addition to their nutritional value, they possess medicinal properties and other beneficial effects and health-promoting effects.* Pleurotus* species have been used by human cultures all over the world for many years [17, 85–89].

These species have been used as medicinal mushrooms for long time since they contain several compounds with important pharmacological/nutraceutical properties. Some of these substances are lectins with immunomodulatory, antiproliferative, and antitumor activities; phenolic compounds with antioxidant activities; and polysaccharides (polysaccharopeptides and polysaccharide proteins) with immunoenhancing and anticancer activities. *β*-glucans isolated from* Pleurotus pulmonarius* demonstrated an anti-inflammatory response in rats with colitis, and* P. ostreatus* inhibited leukocyte migration to acetic acid-injured tissues. An extract from* P. florida* suppressed inflammation.* Pleurotus* has also been reported with hematological, antiviral, antitumor, antibacterial, hypocholesterolic and immunomodulatory activities, and antioxidant properties [17, 86, 90–94].

Maity et al. [95] reported the stimulation of macrophages with different concentrations of the heteroglycan isolated from* P. ostreatus,* and Lavi et al. [87] and Tong et al. [96] reported antiproliferative and proapoptotic effects on colon cancer cells from an aqueous polysaccharide extract. In addition, Jedinak et al. [91] concluded that the edible oyster mushroom may be considered a functional food due to its anti-inflammatory activity and potential to control inflammation. Moreover,* P. ostreatus* exhibits hypocholesterolemic effect on rats with normal cholesterolemia or hypercholesterolemia and hereditary cholesterol disorders [97]. Other authors reported some species of* Pleurotus* with this hypocholesterolemic effect as well [3]. According to Manzi and Pizzoferrato [50],* Pleurotus pulmunarius* apparently seems to be the richest source of fungal *β*-glucans. They also concluded that *β*-glucans in mushrooms are distributed in the soluble and insoluble dietary fraction.

*P. citrinopileatus, P. djamor, P. eryngii, P. flabellatus, P. florida, P. ostreatus*, and* P. sajor-caju* were evaluated by Mishra et al. [88]. The authors concluded that* P. eryngii* had the highest contents of phenolics, followed by* P. djamor.* Besides,* P. eryngii* had a better antioxidant activity and* P. citrinopileatus* had more ascorbic acid and chelating activity.

Kanagasabapathy et al. [92] reported antitumor effects and antioxidant properties by* P. sajor-caju*. The aqueous and butanol extracts exhibited the highest antioxidant activity and corresponded to the total phenolic content. Also, a ribonuclease from* P. sajor-caju* presented antimicrobial, antimutagenic, and antiproliferative activities. However, the antiproliferative activity of this fungus may result from its specific proteins, terpenoids, steroids, fatty acids, and phenolic compounds [98]. On the other hand, Finimundy et al. [17] reported evidence that* P. sajor-caju* is a potential source of antioxidant and anticancer compounds.

Water-soluble polysaccharides extracted from* P. tuber-regium*, a novel edible mushroom, showed effective antiproliferative activity against human leukemia cells and induced apoptosis in HL-60 cells [5, 99]. Besides, Li et al. [100] isolated a potent lectin from* P. citrinopileatus* with antitumor activity in mice sarcoma.

*Pleurotus giganteus* is a culinary mushroom with outstanding sensory properties. It contains 15.4 g of protein and 33.3 g of dietary fiber/100 g of mushroom (dry weigh basis) and it also has important amounts of carbohydrates. It is rich in minerals like magnesium (67.64 mg/100 g dry weight) and potassium (1,345.7 mg/100 g dry weight). Its carbohydrate content is 4 to 11-times higher than other edible mushrooms [101]. The aqueous and ethanolic extracts from* P. giganteus* have shown antioxidant, genotoxic, and liver protective properties and have a high effect on neuronal differentiation and neurite outgrowth. The high potassium level in the fruiting bodies and the presence of bioactive compounds, mainly triterpenoids, could be responsible for the neuroactivity [101, 102].

#### 2.3.4. Ganoderma

**Figure 4:**
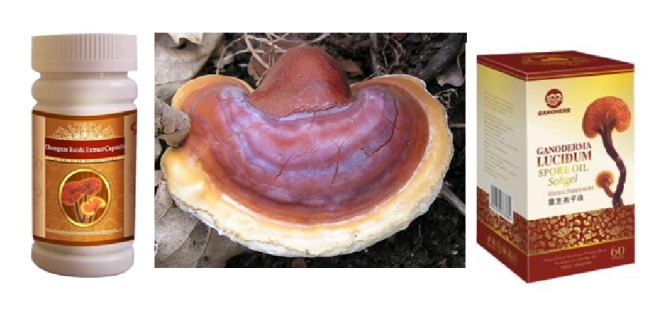
*Ganoderma* the “mushroom of immortality.”

The “mushroom of immortality,” commonly known as Lingzhi or Reishi, has been used in traditional Chinese medicine to improve health and longevity for thousands of years, as well as in the treatment of neurasthenia, hypertension, hepatopathy, and carcinoma (Figure 4). It is one of the most popular medicinal mushrooms in China, Japan, and Korea. It has been under modern biochemical and pharmacological research during the last decades [103, 104]. Modern pharmacological tests have also demonstrated some important characteristics of this fungus, such as immunomodulating, antiallergic, antiradiation, antitumor, anti-inflammatory, antiparasitic, and antioxidant properties. Some benefits for the cardiovascular, respiratory, endocrine, and metabolic systems have also been described [40, 105, 106].

In Asia,* Ganoderma* has been administered for centuries as treatment for cancer; it exhibits anticancer effect alone or in combination with chemotherapy and radiotherapy.* Ganoderma* decreases viability of human cancer cells, induces cell apoptosis, inhibits cell proliferation, suppresses the motility of invasive breast and prostate cancer cells, and prevents the onset of various types of cancer [107–111]. Also, Chen and Zhong [112] reported the inhibition of tumor invasion, metastasis and cell adhesion, promotion of cell aggregation, and suppression of cell migration in human colon tumor cell lines. Additionally, Ye et al. [113] reported antitumor action* in vitro* against mouse lymphocytic leukemia, and Lai et al. [114] reported the suppression of epidermoid cervical carcinoma. Water-soluble polysaccharides from* Ganoderma* act over more than 20 types of cancer and strongly inhibit tumor growth [106].

The major biologically active polysaccharides from* Ganoderma* are *β*-glucans, and the anticancer and antimetastatic activities are due to its polysaccharides and triterpenoid components. These compounds may be associated with their immunostimulating activities and antioxidant capacity. It also contains a large number of proteins and peptides with biological activities, such as lectins, ribosome inactivating proteins, antimicrobial proteins, ribonucleases, and laccases, which are important for life activity and show immunomodulatory and antitumor effects as well [39, 40, 52, 104, 106, 115].

*Ganoderma* presents three characteristics for prevention or treatment of diseases. First, it does not produce any toxicity or side effects; second, it does not act on a specific organ; and third, it promotes the improvement of normalization of the organ function. Modern pharmacological and clinical trials have demonstrated that this fungus shows a significant effect on the prevention and treatment of various diseases, especially cancer, including immunomodulation, induction of cytokine production, antiallergic, antiradiation, antitumor, anti-inflammatory, antiparasitic, and antioxidant effects, as well as benefits for the cardiovascular, respiratory, endocrine, and metabolic systems [40, 104–106].

A large collection of scientific information on bioactive components and pharmacological properties, mainly on the anticancer potential of* Ganoderma*, is available; it is focused on the anticancer effect, regulation of cell cycle, and cell signaling [52, 103, 106, 116–120]. Moreover, Weng and Yen [115] studied the inhibitory activity against invasive and metastatic behaviors (*i.e.*, adhesion, migration, and angiogenesis) in various cancer cells* in vitro* or implanted in mice.

Nowadays,* Ganoderma* is recognized as an alternative adjuvant in the treatment of leukemia, carcinoma, hepatitis, and diabetes, as well as an immune system enhancer with health benefits. In general, it is safe to be used for a long period of time [104]. The dried powder and aqueous/ethanol extracts of* G. lucidum* are used worldwide as dietary supplement [121]. Boh [122] studied around 270 patents for fruit bodies and mycelia cultivation methods of* Ganoderma lucidum*, basidiomycete mushroom with strong anticancer effects. Boh concluded that the anticancer activity of this fungus may be attributed to at least five groups of mechanisms: (1) activation/modulation of the immune response of the host, (2) direct cytotoxicity to cancer cells, (3) inhibition of tumor-induced angiogenesis, (4) inhibition of cancer cells proliferation and invasive metastasis behaviour, and (5) carcinogens deactivation with protection of cells.

#### 2.3.5. Huitlacoche

*U. maydis* belongs to the Ustilaginales order that includes semiobligate biotrophic plant pathogenic fungi that infects only maize and its progenitor plant teosinte (*Zea mays*). It is a heterothallic fungus with a dimorphic life cycle, saprophytic and a parasitic phase; in nature, the pathogenic and sexual development are inseparable. Also,* U. maydis* has been established as a robust pathogenic model for studying fungi and fungi-plant relationships, especially because the morphological transitions throughout its life cycle, easy culture, genetic manipulation in the laboratory, mating type, biotrophic host interaction, genetic properties to elucidate the molecular mechanisms of the interaction between plant and pathogen, and the severe disease symptoms that it induces in infected maize. On the other hand,* U. maydis* is responsible for the corn smut, characterized by the formation of galls or tumors, mainly in ears. These ear galls have been used as food in Mexico since pre-Columbian times [123].

**Figure 5:**
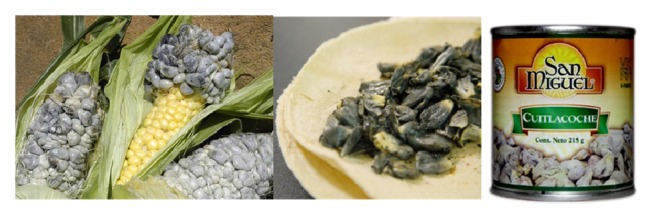
Huitlacoche, the corn smut caused by the fungus* Ustilago maydis* on maize.

Cuitlacoche or huitlacoche is the Aztec name given to these young, fleshy, and edible galls (Figure 5). In Mexico, it has been traditionally prized and many hundreds of tons of fresh, prepared, or processed huitlacoche are sold annually. Nowadays, it is a culinary delight for international chefs and has been accepted as a food delicacy in several countries and introduced into countless worldwide markets in countries like Japan, China, and some of the European Community, as France, Spain, and Germany. Also, in the United States there has been a great interest to produce huitlacoche due to an emerging acceptance by the North American public, who noticed it as a gourmet food and now can be purchased on the Internet at high prices. In addition to its unique flavor, huitlacoche has been identified as a high-quality functional food and could be included into the daily diet for its attractive characteristics, selected nutrients, valuable compounds, and nutraceutical potential [123].

The nutritional value of this mushroom has great importance for human diet. The protein content of huitlacoche varies from 9.7 to 16.4% (wet basis) and it is similar or sometimes superior to other edible mushrooms and definitely superior to the maize protein content (10%). Therefore, huitlacoche may be proposed as an alternative protein source for vegetarian diets in the same way as other edible mushrooms have been suggested. Huitlacoche contains almost all essential amino acids, lysine (6.3–7.3 g/100 g protein) being one of the most abundant. Other abundant amino acids include serine, glycine, aspartic and glutamic acid, which collectively account for 44.3 to 48.9% of total amino acids. The high content of essential fatty acids also suggests an interesting nutritional value for huitlacoche; some important fatty acids are oleic and linoleic acids (54.5 to 77.5%) [124, 125].

Huitlacoche produced under different conditions had high concentrations of selected nutrients and compounds with nutraceutical potential, which showed variations due to maize genotype, stage of development, and cooking process. Valdez-Morales et al. [126] identified eight monosaccharides and eight alditols in huitlacoche; glucose and fructose were the most abundant, constituting approximately 81% of the total carbohydrates. Galactose, xylose, arabinose, and mannose were found in lesser proportions. Glycerol, glucitol, and mannitol were the most representative alditols. Also, huitlacoche contains, within its dietary fiber, homoglycans and heteroglycans, similar to those found in other edible mushrooms (Table 4).

The content of *β*-glucans in huitlacoche is higher (20–120 mg/g of huitlacoche, in dry weight) than that reported for corn (0.5–3.8 mg/g) and similar to other edible mushrooms [126]. *β*-glucans activates the complement and improve the response of the macrophages and killer cells. They can also be antioncogenic due to their protector effect against genotoxic compounds and because of their antiangiogenic effect. These authors also analyzed different maize genotypes to produce huitlacoche and found differences in *β*-glucans concentrations and concluded that creole corn showed the highest amounts; this maize was proposed for huitlacoche production in Mexico. Besides, they concluded that the amount of *β*-glucans in huitlacoche is higher than that reported in corn and it is similar to other edible mushrooms.

The search for medicinal substances from fungi has become a matter of great interest. It has been confirmed that higher basidiomycetes contain bioactive substances that possess hyperlipidemic, antitumoral, immunomodulating, anti-inflammatory, antimutagenic, antiatherogenic, hypoglycemic, and other health-promoting properties. Valdez-Morales et al. [126] also reported antimutagenic capacity (41.0 to 76.0%) in huitlacoche, but without assessing the compounds that confer this activity. They also revealed that the total phenol concentration in huitlacoche is elevated and within that reported for other edible fungi (Table 5).

Huitlacoche has been characterized as a high-quality nutraceutical food as well as an attractive ingredient to enrich other dishes, mainly for its extraordinary flavor and exceptional quality. The introduction of this food into the international market requires the development of techniques for massive production during the whole year, particularly because this parasitic fungus only grows in maize ears. An efficient method to inoculate maize plants with* U. maydis* began in the 18th century when it was unsuccessfully attempted to demonstrate the causal relationship between common smut and maize. Many studies have been focused on ear infection, and the most important finding was observed in the silk-channel inoculation procedure, resulting with a much higher incidence of ear galls than natural infection [125]. However, many factors are involved in this process and the efficient production of huitlacoche by inoculating silks with* U. maydis* may require accurate timing of inoculation and control of pollination to maximize the number of kernels infected and the huitlacoche yield.

### 2.4. Other Mushrooms

Some other species of mushrooms are also edible and possess health benefits.* Trametes versicolor* has been shown to promote chemopreventive potential; it inhibits growth of several human cancer cell lines, acts as adjuvant in breast cancer prevention and has a significant IC_50_ value [127, 128].

*Grifola frondosa* is promoted as anticancer agent, particularly on human gastric carcinoma, such effect results from the induction of cell apoptosis and could significantly accelerate the anticancer activity [129, 130].

In this context, it could be mentioned that* Cordyceps militaris* has several beneficial effects and it is used for multiple medicinal purposes. It acts as an antitumor, antiproliferative, antimetastatic, insecticidal, and antibacterial compound. More than 21 clinically approved beneficial effects for human health have been found in this mushroom [131, 132]. Extracts of* C. militaris* have been used for its immnunomodulatory and anti-inflammatory effects. Besides, it is also a cancer preventive material and is effective against chronic bronchitis, influenza A, and viral infections [133].

*Cordyceps sinensis* contains substances called cordycepin, cordycepic acid, with therapeutic applications like the effects of increased oxygen utilization, ATP production, and stabilization of blood sugar metabolism. Besides, it has antibacterial function, reduces asthma, and lowers blood pressure. On the other hand, it has been reported as organ protector, as well as with a protective effect for heart, liver, and kidney diseases. Also,* C. sinensis* has sedative effect on the central nervous system [134].

*Antrodia cinnanomea* is a medicinal mushroom native to Taiwan with various functional compounds and a total of 105 Taiwan patent applications. Different commercial products are made with this mushroom and it has been used to treat food and drug intoxication, diarrhea, abdominal pain, hypertension, skin itching, and cancer [135].

*Panellus serotinus* (Mukitake) is extremely appreciable in Japan as one of the most delicious edible mushrooms. The use of this fungus helps to prevent the development of nonalcoholic fatty liver disease [136].

Most* Auricularia* species are edible and are grown commercially in China*. A. polytricha* has potential medicinal properties and is considered effective to reduce LDL cholesterol and aortic atherosclerotic plaque; it also has antitumor and anticoagulant activities. Besides,* A. auricula-judae* is a popular ingredient in many Chinese dishes; it has been used as a blood tonic and has shown antitumor, hypoglycemic, anticoagulant, and cholesterol-lowering properties [137, 138].

*Flammulina velutipes* is available as fresh or canned product and it is traditionally used for soups in China. It contains biologically active components such as dietary fiber, polysaccharides, and antioxidants, which reduce blood sugar, blood pressure, and cholesterol [139].

## 3. Conclusions

Several mushroom species have been pointed out as sources of bioactive compounds, in addition to their important nutritional value. The inclusion of whole mushrooms into the diet may have efficacy as potential dietary supplements.

The production of mushrooms and the extraction of bioactive metabolites is a key feature for the development of efficient biotechnological methods to obtain these metabolites. It has been shown by a wide range of studies that mushrooms contain components with outstanding properties to prevent or treat different type of diseases.

Powder formulations of some species have revealed the presence of essential nutrients. They present a low fat content and can be used in low-calorie diets, just like the mushrooms fruiting bodies. Some formulations could be used as antioxidants to prevent oxidative stress and thus ageing.

Future studies into the mechanisms of action of mushroom extracts will help us to further delineate the interesting roles and properties of various mushroom phytochemicals in the prevention and treatment of some degenerative diseases.

In view of the current situation, the research of bioactive components in edible wild and cultivated mushrooms is yet deficient. There are numerous potential characteristics and old and novel properties, provided by mushrooms with nutraceutical and health benefits, which deserve further investigations.

## Figures and Tables

**Table 1:** Proximal composition of some edible mushrooms (dry basis).

Species	Protein	Fat	Ash	Carbohydrates	Energy
	%	%	%	%	kcal/kg
*Agaricus bisporus *	14.1	2.2	9.7	74.0	325
*Lentinus edodes *	4.5	1.73	6.7	87.1	772
*Pleurotus ostreatus *	7.0	1.4	5.7	85.9	416
*Pleurotus eryngii *	11.0	1.5	6.2	81.4	421
*Pleurotus sajor-caju *	37.4	1.0	6.3	55.3	
*Pleurotus giganteus *	17.7	4.3	—	78.0	364
Dry powder formulations					
*Agaricus blazei *	31.3	1.8	7.5	59.4	379
*Lentinus edodes *	12.8	1.0	4.3	81.9	388

Adapted from Carneiro et al. 2013 [22]; Kalač 2013 [29]; Phan et al. 2012 [101]; Reis et al. 2012 [30].

**Table 2:** Composition of sugars of some edible mushrooms (dry weight).

Species	Fructose	Mannitol	Sucrose	Trehalose	Total sugars
	(g/100 g fresh weight)				
*Agaricus bisporus *	0.03	5.6	nd	0.16	5.79
*Lentinus edodes *	0.69	10.01	nd	3.38	14.03
*Pleurotus ostreatus *	0.01	0.54	nd	4.42	4.97
*Pleurotus eryngii *	0.03	0.60	0.03	8.01	8.67
Dry powder formulations					
*Agaricus blazei *	0.27	60.89	nd	5.74	66.91
*Lentinus edodes *	nd	23.3	nd	13.22	38.31

Adapted from Carneiro et al. 2013 [22]; Reis et al. 2012 [30]. Nd, not detected.

**Table 3:** Fatty acids content of some edible mushrooms.

Species	Fatty acid (g/100 g fresh weight)				
	Palmitic (C16:0)	Stearic (C18:0)	Oleic (C18:1)	Linoleic (C18:2)	Linolenic (C18:3)
*Agaricus bisporus *	11.9	3.1	1.1	77.7	0.1
*Lentinus edodes *	10.3	1.6	2.3	81.1	0.1
*Pleurotus ostreatus *	11.2	1.6	12.3	68.9	0.1
*Pleurotus eryngii *	12.8	1.7	12.3	68.8	0.1
Dry powder formulations					
*Agaricus blazei *	11.38	2.8	1.85	72.42	nd
*Lentinus edodes *	11.78	1.09	3.28	78.59	0.59

Adapted from Carneiro et al. 2013 [22]; Reis et al. 2012 [30]. Nd, not detected.

**Table 4:** Dietary fiber fractions, *β*-glucans, and free sugars in huitlacoche (dry basis).

Component	Units
Dietary fiber	% total content

Total dietary fiber	39–60
Soluble dietary fiber	9–29
Insoluble dietary fiber	22–51

	mg/g huitlacoche

*β*-glucans	20–120
Total free sugars	56–267
Glucose	53–231
Fructose	19–138
Galactose	0.2–3.5
Arabinose	0.2–3.3
Mannose	0–1.8
Xylose	0–2

Adapted from Valdez-Morales et al. (2010) [126].

**Table 5:** Phenolic compounds of huitlacoche from creole Mexican maize.

Phenolic compound	*μ*g/g huitlacoche (dry basis)
Gallic acid	2.4–2.6
Ferulic acid	514.1–544.2
Caffeic acid	26.3–27.4
*p*-Coumaric acid	10.2–10.6
*o*-Coumaric acid	4.4–4.8
Rutin	6.2–6.4
Catechin	11.0–11.7
Quercetin	42.4–45.2
Total phenols	636.8–667.4

Adapted from Valdez-Morales et al. (2010) [126].
